# Current status of phosphine resistance in Indian field populations of *Tribolium castaneum* and its influence on antioxidant enzyme activities

**DOI:** 10.1038/s41598-023-43681-y

**Published:** 2023-10-01

**Authors:** Satyapriya Singh, Suresh M. Nebapure, Sukumar Taria, Doddachowdappa Sagar, Sabtharishi Subramanian

**Affiliations:** 1 Division of Entomology, ICAR-Indian Agricultural Research Institute, New Delhi, 110012 India; 2 Division of Plant Physiology, ICAR-Indian Agricultural Research Institute, New Delhi, 110012 India

**Keywords:** Entomology, Biochemical assays

## Abstract

Resistance to phosphine is widely reported in several stored product insect pests globally. However, knowledge of its prevalence and the association of antioxidant enzymes with phosphine resistance is limited. Herein, we assessed the levels of phosphine susceptibility and estimated the antioxidant enzyme activities viz., superoxide dismutase (SOD), peroxidase (POX), and catalase (CAT) in selected Indian populations of red flour beetle *Tribolium castaneum* (Herbst). Dose–response probit assays revealed that the LC_50_ values ranged from 0.038 to 1.277 mg L^−1^ showing 2.11 to 70.94-fold resistance to phosphine compared to susceptible check. Activities of antioxidant enzymes varied significantly between the *T. castaneum* populations following phosphine exposure. The magnitude of SOD activity ranged from 8.77 to18.82 U mg^−1^ protein, while, the activities of POX and CAT varied between 52.42 and 408.32 and 61.11 to 247.49 µM H_2_O_2_ reduced min^−1^ mg^−1^ of protein, respectively. The correlation analysis revealed a significant positive association of SOD (r = 0.89) and POX (r = 0.98) with increased resistance ratio, while the CAT (r = − 0.98) is negatively linked with resistance to phosphine. A principal component analysis identified phosphine resistance was closely associated with POX and SOD activities but was unrelated to the CAT activity. Our results throw light on the varied association of antioxidant enzyme activities in response to phosphine fumigation in field populations of *T. castaneum*. Further studies on the biochemical and molecular basis of phosphine stress in insects may help to devise suitable strategies to safeguard storage commodities and ensure a sustainable environment.

## Introduction

Food grains are more prone to biotic stresses in stored conditions. Insects are one of the prime causes of deterioration in quality and quantity in post-harvest storage. The damage losses are estimated to account for approximately 5–10% and 20–30% in temperate and tropical countries worldwide, respectively^1^.The red flour beetle, *Tribolium castaneum* (Herbst) (Coleoptera,Tenebrionidae), is an important secondary pest of stored agricultural commodities wrecking damage to flour, cereal, beans, and nuts^2^. Apart from direct losses, this beetle secrets benzoquinones (viz., a carcinogenic substance with persistent and objectionable odour) that influence characteristic colour change of the flour and cause severe health risks to human consumption^3^. Thus, *T. castaneum* exaggerates both the quality and quantity of food products.

Phosphine is an eco-friendly and economically affordable fumigant used worldwide for management of stored product insect pests. However, prolonged use of the fumigant and adoption of inadequate fumigation practices have resulted in development of resistance to this fumigant by several stored product insect pests^4,5^. Non availability of suitable alternative fumigant makes phosphine inevitable in control of storage pests in bulk grain storages.

Studies have documented strong resistance to phosphine in *T. castaneum* populations from USA^6,7^, Australia^8^, Turkey^9^, and Greece^10^. The improper and widespread use of phosphine has led to the build-up of resistance levels in several stored insect species^5^. The increasing levels of resistance warrant increased usage of fumigants which have raised concerns about safety storage of food grains. Additionally, insect resistance contributes to economic damage affecting the grain's market price, nutritional efficiencies and poses threat to global food security^11^.

Stored grain insect possesses both ‘strong’ and ‘weak’ resistance to phosphine, particularly in *T. cataneum* and *R. dominica*^8^. The detailed genetic study of these two species identified two loci (*rph1* and *rph 2*) conferring the resistance development^8,12^. The gene for strong resistance was identified as the metabolic enzyme dihydrolipoamide dehydrogenase (DLD) in *T. cataneum* and *R. dominica*^13^*.* Certain point mutations in DLD are associated with strong resistance to phosphine in insects^13^.

Being heterothermic organisms, insects have evolved with various adaptations to avoid phosphine toxicity^14^. As respiratory poison, phosphine toxicity inferred oxidative damage and oxidative stress resulting in aerobic cellular metabolism, which implies producing pro-oxidant substances, viz*.,* reactive oxygen species (ROS)^15^. ROS alters cells function by damaging the oxidation of nuclear and mitochondrial DNA, lipids, and proteins^15^. However, insects counteract the deleterious effect of ROS through the induction of antioxidant systems to maintain physiological homeostasis^16^. The antioxidant enzymes include superoxide dismutase (SOD), catalase (CAT), and peroxidase (POX)^17^. The SOD helps in the conversion of superoxide anion (O_2_^**·−**^) into oxygen (O_2_) and hydrogen peroxide (H_2_O_2_). Further, CAT and POX dissociate H_2_O_2_ into water (H_2_O) and oxygen (O_2_)^18^.

Regularly monitoring phosphine resistance in stored commodities insects is indispensable for ensuring its use’s sustainability and food security in the long run^6^. Further, the prevalence of phosphine resistance is hasty and staggered in *T. castaneum* globally. Although resistance to phosphine is granted a significant issue, detailed information about the level of phosphine resistance is scanty. Nonetheless, little is known about the variability of antioxidant defences to counteract the ROS for building tolerance to phosphine toxicity in *T. castaneum*. Hence, we conducted studies to assess the phosphine susceptibility in field populations of *T. castaneum* and examined its association with antioxidant enzyme activities, viz., SOD, POX, and CAT using appropriate statistical tools such as Pearson’s Correlation matrix and Principal Component Analysis. The outcome of this would help in increasing our understanding on the oxidative stress associated with phosphine tolerance in insects and may aid in developing sustainable management practices for this cosmopolitan stored pest.

## Materials and methods

The present experiment is conducted at the Division of Entomology, ICAR- Indian Agricultural Research Institute, New Delhi (28.6418N, 77.1695E, 246.63 ± 6 m).

### Insect populations and culture

**Figure 1:**
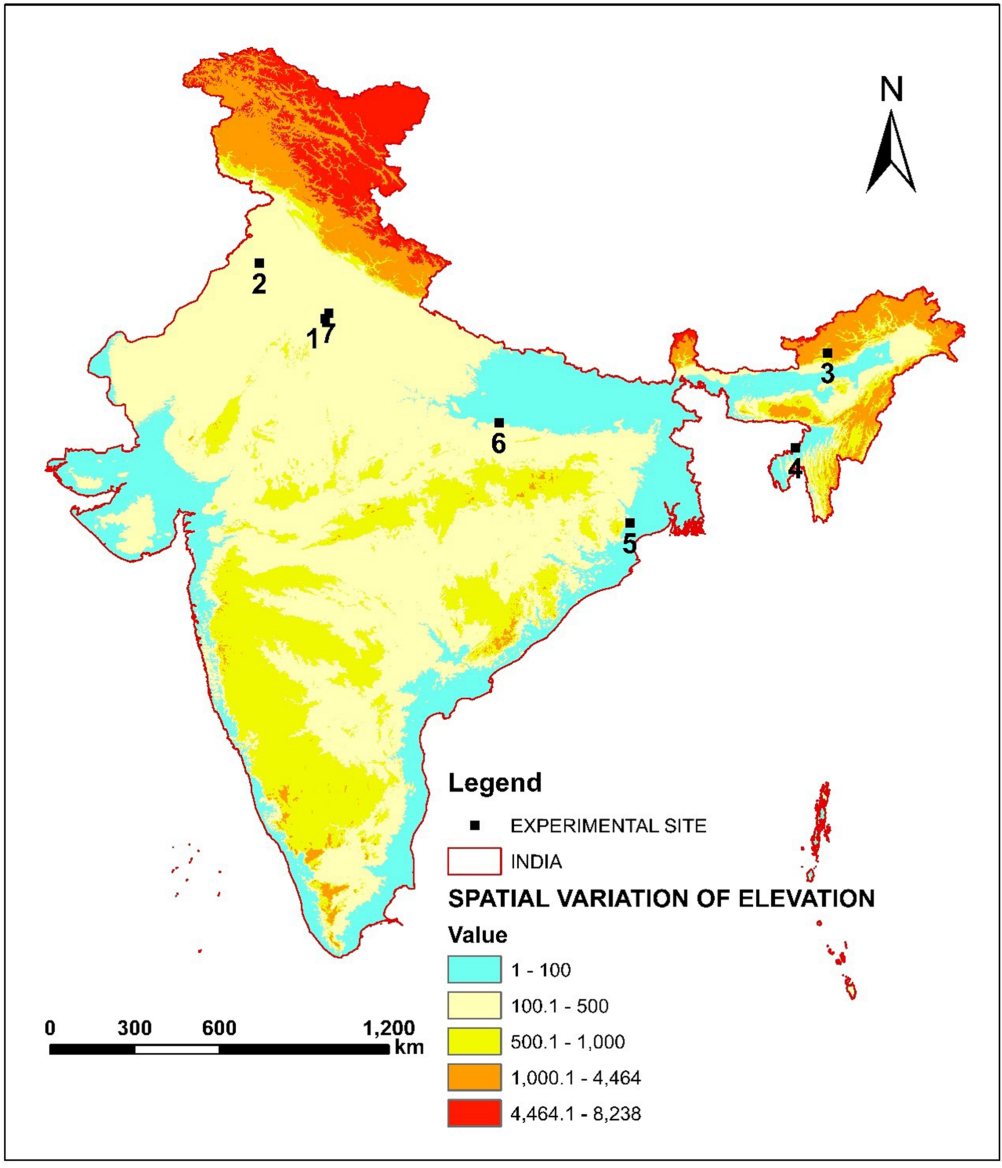
Location map of the *T. castaneum* collection sites. Location map of the study area. Collection sites are indicated in numerical letters and markings (1: Gurgaon, 2: Malwinder Singh, 3: East Kameng, 4: Kailashahar, 5: Mayurbhanj, 6: Mirzapur, 7: Lab susceptible); This map was prepared using the software, ArcGIS version 10.4.

Six populations of *T. castaneum* were collected from different states across India (Table 1; Fig. 1) and one lab population was considered for the susceptible check. The collection areas were grain storage sites and local markets. Insects were collected with their respective hosts. The *Tribolium* population was identified based on morphological parameters (unlike the confused flour beetle, the antennae of the red flour beetle are distinctly clubbed gradually having three-segmented structure) and as well by using mtCO1 markers. The collected populations were separated from the host in the laboratory and reared in 1.5 L glass bottles on a mixture of 95% all-purpose wheat flour and 5% brewer’s yeast (wt:wt). The rearing chamber was maintained at 30 ± 1 °C and 65 ± 5% relative humidity with a photoperiod of 16:8 (L:D).

**Table 1:** Location details of *Tribolium castaneum* (Herbst) collected populations.

Population	Region	Geo-coordinate	Source
Gurgaon	Haryana	28.4575 N, 77.0263 E	Flour (Wheat)
Malwinder Singh	Punjab	30.2110 N, 74.9455 E	Grain storage (Rice)
East Kameng	Arunachal Pradesh	27.3610 N, 93.0401 E	Grain storage (Rice)
Kailashahar	Tripura	24.3268 N, 92.0126 E	Flour (Wheat)
Mayurbhanj	Odisha	21.9330 N, 86.7330 E	Flour (Wheat)
Mirzapur	Uttar Pradesh	25.1337 N, 82.5644 E	Grain storage (Rice)
Lab susceptible	New Delhi	28.6288 N, 77.1471 E	Lab population, Flour (Wheat)

The table provides the details of the collection sites of the *T. castaneum* field populations across India, hosts (source grain/flour) and Geo-coordinates of respective collection sites.

### Phosphine generation and bioassay

Phosphine gas (PH_3_) was generated in a gas burette using the commercial formulation of aluminum phosphide (AL_2_P_3_) tablet viz., 56% (F) (Quickphos*) suspended in 5% sulphuric acid solution^19^. Mortality responses to phosphine were measured against a range of PH_3_ concentrations viz., 0 to 1.5 mg L^−1^, including control. Adult beetles (mixed sexes; two weeks old) were used for bioassay. Adults were kept in plastic vials (30 mL; 4.5 cm height × 1.2 cm diameter). The vials were covered with a white muslin cloth, properly labelled, and transferred to air-tight gas desiccators (phosphine chamber).

There were 30 adults per vial representing one replication. Three replications were maintained for each concentration. The desired amount of phosphine was administered by a gas-tight Hamilton syringe through septa in the lid of the desiccator. After an exposure period of 20 h, the insects were removed from the desiccators and transferred to a fresh vial provided with food. A control set was maintained by injecting air into the desiccator instead of phosphine. The fumigation experiments were conducted at 30 ± 1 °C and 65 ± 5% RH. Mortality was recorded after 14 days from the end of the exposure period. The moribund/immobile insects were considered dead for study. The experiment was done in a completely randomized design with three replications for each population. A laboratory population (susceptible check, maintained up to 30 generations) was kept to calculate the resistance ratio.

### Assessment of resistance and resistance ratio estimation

Adult beetles (14 days old) from laboratory-reared field populations were used to determine the resistance level. The mortality data obtained through bioassays (as mentioned in the previous section) were subjected to probit analysis to determine lethal concentrations viz., LC_50_, LC_90_, and LC_99_^19^. The lethal concentrations of phosphine obtained for the lab population were used to calculate the resistance ratio.

$$\mathrm{Resistance\,Ratio }\left(\mathrm{RR}\right)=\frac{\mathrm{LC}50\mathrm{\,of\,the\,test\,population}}{\mathrm{LC}50\mathrm{\,of\,the\,susceptible\,lab\,population }}\times 100$$

### Enzyme assays

#### Tissue processing

The antioxidant enzyme activities viz., Superoxide Dismutase (SOD), Peroxidase (POX), and Catalase were measured in the field populations of *T. castaneum*^20^. The adult beetles of the test populations were exposed to LC_50_ concentration for 20 h and surviving beetles were used for enzyme assays. The survived beetles were homogenized with 100 µL of 50 mM phosphate buffer (pH 7.0) in microcentrifuge tubes (1.5 mL). Homogenization was done using a motorized pellet pestle (Fisher Scientific) in a mini-cooler. The homogenates were centrifuged at 16,000 rpm for 10 min at 4 °C (Eppendorf, Centrifuge 5425R, Germany). The supernatants were used as enzyme extracts for assays.

#### Enzymatic activities

Catalase activity was measured using the method described by Beer and Sizer^21^ with minor modifications. The activity was measured as a decrease in absorbance at 240 nm for 3 min using H_2_O_2_ (20 mM, final concentration) as the substrate (ε = 39.4 L M^−1^ cm^−1^). Peroxidase activity was determined as described by Armstrong et al.^22^ with minor modifications. 0.1 M concentration of H_2_O_2_ was taken as substrate. Absorbance was recorded at 470 nm for 5 min (ε = 26.6 L M^−1^ cm^−1^). CAT and POX activities were expressed as μmol H_2_O_2_ reduced per minute per milligram of protein (μmol min^−1^ mg^−1^). A solution without enzyme extract was considered blank. SOD activity was assessed by the method described by Kakkar^23^. Briefly, the assay mixture contained sodium pyrophosphate buffer (0.052 M), phenazine methosulphate, nitroblue tetrazolium, reduced nicotinamide adenine dinucleotide, and enzyme lysate. The principle relies on the inhibition of the formation of NADH-phenazine methosulfate-nitroblue tetrazolium formazan. Enzyme activity was measured at 560 nm and expressed in terms of the rate of percentage inhibition. One unit of SOD is defined as the amount of enzyme needed to exhibit 50% dismutation of the superoxide radical. All enzymatic activities were estimated with a UV–VIS EPOCH 2 microplate reader spectrophotometer at 25 °C (BioTek, USA) using microplates (Tarsons).

#### Protein estimation

Total protein concentration was determined using the Bradford method (Bradford 1976)^24^ using bovine serum albumin as standard, and the absorbance was recorded at 595 nm.

### Statistical analysis

The corrected mortality data were subjected to probit analysis (Polo Plus, Ver. 2.0 Leora Software 2002) to determine LC_50_, LC_90_, and LC_99_ values and fiducial limits. The Chi-square test was performed to ascertain the goodness of fit^25^. The insect collection sites are depicted in a map using ArcGIS 10.4 software. Analysis of variance and Duncan’s Multiple Range Tests (DMRT) were conducted using the statistical package of “R” of version 4.1.2. Values are presented in the table as the means of the different locations with standard error of means (SEM). Pearson’s Correlations among the traits were displayed using the “Metan” package. PCA biplots analysis used “FactoMineR” and “Factoextra” packages in R for dimension reduction.

## Results

### Toxicity of phosphine to different populations of *T. castaneum*

**Figure 2:**
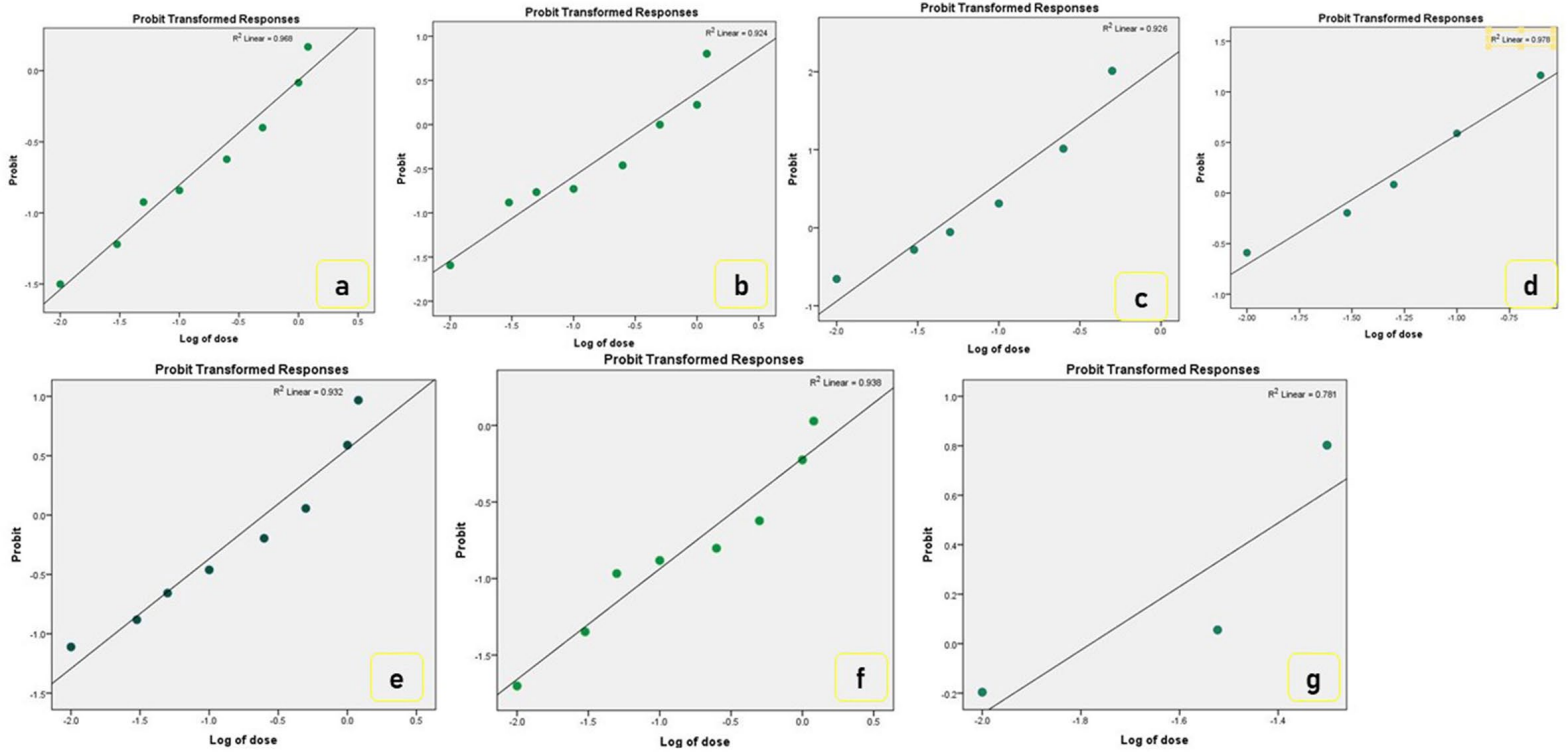
Probit analysis of *T. castaneum* to dose–response mortality. The mortality response of different populations after exposure to phosphine. The dose–response lines of each population were drawn using a probit linear model y = αx + β in which α and β are the slope and intercept, respectively; R^2^ value is depicted. x is the log-transformed dose (mg L^−1^), y is the percent mortality; Locations; (**a**) Gurgaon, (**b**) Malwinder Singh, (**c**) East Kameng, (**d**) Kailashahar, (**e**) Mayurbhanj, (**f**) Mirzapur, (**g**) Lab susceptible.

The collection sites of all *T. castaneum* populations used in this study are shown in Fig. 1. The bioassay study was conducted to generate dose–response data for the *T. castaneum* against phosphine. The results of the dose–response regressions analyzed by probit assay were shown in Table 2. The mortality data obtained for different *T. castaneum* populations when exposed to a range of PH_3_ concentrations for 20 h at 30 °C, revealed substantial variation in susceptibility to phosphine in these populations as compared to the susceptible lab population. The calculated χ^2^ value was lesser than the table value (χ^2^ = 14.067, *df* = 7) at a 5% significance level, symbolizing population homogeneity (Table 2). Henceforth, the probit model was considered to be appropriate. The tested *T. castaneum* populations exhibited varying levels of slopes for probit response curves (0.961 to 1.645) for phosphine. The susceptibility of the populations (LC_50_) ranged from 0.038 to 1.277 mg L^−1^ (Table 2). The Mirzapur population was the least susceptible to phosphine fumigation, with an LC_50_ value of 1.227 mg L^−1^, compared to the lab population with an LC_50_ value of 0.018 mg L^−1^. Two *T. castaneum* populations from Kailashahar and East Kameng were highly susceptible to phosphine with the LC_50_ values being 0.038 mg L^−1^ and 0.043, respectively. Comparatively, populations from Malwinder Singh and Gurgaon were found to be lower susceptibility with LC_50_ values of 0.825 and 0.881 mg L^−1^, respectively. Similarly, the LC_90_ and LC_99_ values ranged from 0.075 to 27.546 mg L^−1^ and 0.239 to 336.96 mg L^−1^ across the population (Table 2). Moreover, the dose–response curve depicts the percentage of mortality across the population to phosphine exposure (Fig. 2). There was upto thirty-four-fold increase in resistance ratio in Mirzapur with comparison to the most susceptible population viz., Kailashahar-2.11%. However, the magnitude of resistance was recorded as 70.94-fold in comparison to the lab population.

**Table 2:** Monitoring phosphine resistance in field-collected populations of *T. castaneum* from India.

Population	LC_50_ (mg L^−1^) (95% Fiducial limit)	LC_90_ (mg L^−1^) (95% Fiducial limit)	LC_99_ (mg L^−1^) (95% Fiducial limit)	Slope ± SE	*df*	*Chi-square*	Heterogeneity	RR (%)	*p*-value
Gurgaon	0.881 (0.571–1.416)	17.210 (6.979–108.41)	194.19 (42.722–4684.7)	0.993 ± 0.133	6	14.286	2.041	48.94	0.423
Malwinder Singh	0.825 (0.456–1.410)	11.436 (4.450–168.463)	97.546 (19.235–12,326.19)	1.122 ± 0.169	6	21.899	3.128	45.83	0.432
East Kameng	0.043 (0.032–0.057)	0.294 (0.211–0.458)	1.402 (0.820–3.017	1.541 ± 0.102	6	16.771	2.396	2.39	0.402
Kailashahar	0.038 (0.028–0.050)	0.230 (0.172–0.334)	0.991 (0.619–1.933)	1.645 ± 0.125	6	12.632	1.805	2.11	0.448
Mayurbhanj	0.296 (0.091–0.492)	2.775 (1.537–13.032)	17.206 (5.544–525.182)	1.319 ± 0.186	6	26.107	3.730	16.44	0.500
Mirzapur	1.277 (0.794–2.478)	27.546 (9.199–322.765)	336.96 (56.704–20,439.00)	0.961 ± 0.132	6	16.675	2.382	70.94	0.504
Lab susceptible	0.018 (0.012–0.024)	0.075 (0.053–0.131)	0.239 (0.135–0.700)	2.070 ± 0.198	6	23.309	3.330	1.00	0.314

Probit mortality and dose–response activity of six field populations of *T. castaneum* collected from different locations. LC_50_, median lethal concentration (concentration in mg L^−1^ that would kill 50% of the treated population), similarly LC_90_ and LC_99_ (concentration in mg L^−1^ that would kill 90% and 99% of the treated population, respectively). The fiducial limit is presented at the 95% confidence level; total 720 numbers of insect was taken for each population with eight doses; chi-square value at 95% confidence level; RR, resistance ratio (RR of a test population = (LC_50_ of the test population/LC_50_ of the susceptible lab population × 100); *df* degrees of freedom (i.e., df = n − 2, where n is the number of concentrations administered for bioassay); ‘p-value at 0.05% level of significance.

### The specific activity of antioxidant enzymes in different *T. castaneum* populations

#### Superoxide dismutase activity

The activity of SOD was induced following phosphine exposure. The activity ranged from 8.77 to 18.82 U mg^−1^ protein, with a significant level of variation (F value = 72.66, *df* = 6, *p* < 0.001) across the population (Table 3). The study revealed that the Mirzapur population showed the highest SOD activity (18.82 U mg^−1^ protein) compared to the laboratory-susceptible population (5.00 U mg^−1^ protein). Gurgaon and Malwinder Singh populations exhibited relatively lower activity*,* 17.16 and 15.99 U mg^−1^ protein respectively. The SOD parameter was found to be 12.84, 10.66, and 8.77 U mg^−1^ protein in Kailashahar, Mayurbhanj, and East Kameng compared to the reference lab population, respectively.

**Table 3:** Specific activities of antioxidant enzymes in different *T. castaneum* (Herbst) population.

Population	Catalase (µM min^−1^ mg^−1^ of protein)	Peroxidase (µM min^−1^ mg^−1^ of protein)	SOD (U mg^−1^ protein)
Gurgaon	146.17 ± 5.220^c^	258.68 ± 9.291^b^	17.16 ± 0.09^ab^
Malwinder Singh	143.33 ± 19.140^c^	232.74 ± 17.184^b^	15.99 ± 0.08^b^
East Kameng	217.77 ± 14.568^ab^	82.19 ± 3.208^c^	8.77 ± 0.09^e^
Kailashahar	231.28 ± 30.884^ab^	86.73 ± 18.431^c^	12.84 ± 0.22^c^
Mayurbhanj	193.40 ± 19.140^b^	106.81 ± 9.387^c^	10.66 ± 0.09^d^
Mirzapur	61.11 ± 22.885^d^	408.32 ± 31.500^a^	18.82 ± 0.02^a^
Lab susceptible	247.49 ± 31.402^a^	52.42 ± 3.492^a^	5.00 ± 0.51^f^
df	6	6	6
F value	26.566	180.294	72.666
P value	*p* < 0.001	*p* < 0.001	*p* < 0.001

Specific activities of antioxidant enzymes in different field populations of *T. castaneum*. The values of mean ± standard error followed by distinct letters are significantly different (p < 0.05). One unit of SOD is defined as the amount of enzyme needed to exhibit 50% dismutation of the superoxide radical. The treatment means were separated by Duncan’s multiple range test. Three replications of each enzyme (catalase, peroxidase, and SOD) were carried out to determine the activity presented in the table.

#### Peroxidase activity

The specific activity of Peroxidase differed significantly among the populations of *T. castaneum* (F value = 180.294, *df* = 6, *p* < 0.001), with strength ranging from 82.19 to 408.320 µM H_2_O_2_ reduced min^−1^ mg^−1^ of protein (Table 3). The East Kameng and Kailashahar populations had shown lesser peroxidase activity, i.e., 82.192 and 86.737 µM min^−1^ mg^−1^, respectively. The highest peroxidase activity was observed in the Mirzapur population (408.320 µM min^−1^ mg^−1^) in comparison to the lab-susceptible population (52.42 µM min^−1^ mg^−1^).

#### Catalase activity

A significant difference prevailed in catalase activity across collected *T. castaneum* populations (F value = 26.566, *df* = 6, *p* < 0.001), (Table 3). The activity ranged from 61.11 to 231.28 µM H_2_O_2_ reduced min^−1^ mg^−1^ of protein. Catalase activity escalated in the northeast populations of Kailashahar (231.28 µM min^−1^ mg^−1^) and East Kameng (217.77 µM min^−1^ mg^−1^) in comparison to lab susceptible check (247.49 µM min^−1^ mg^−1^). On the contrary, we observed the lowest activity value in the Mirzapur population (61.11 µM min^−1^ mg^−1^).

#### Correlation of phosphine toxicity with antioxidant enzyme activities

**Figure 3:**
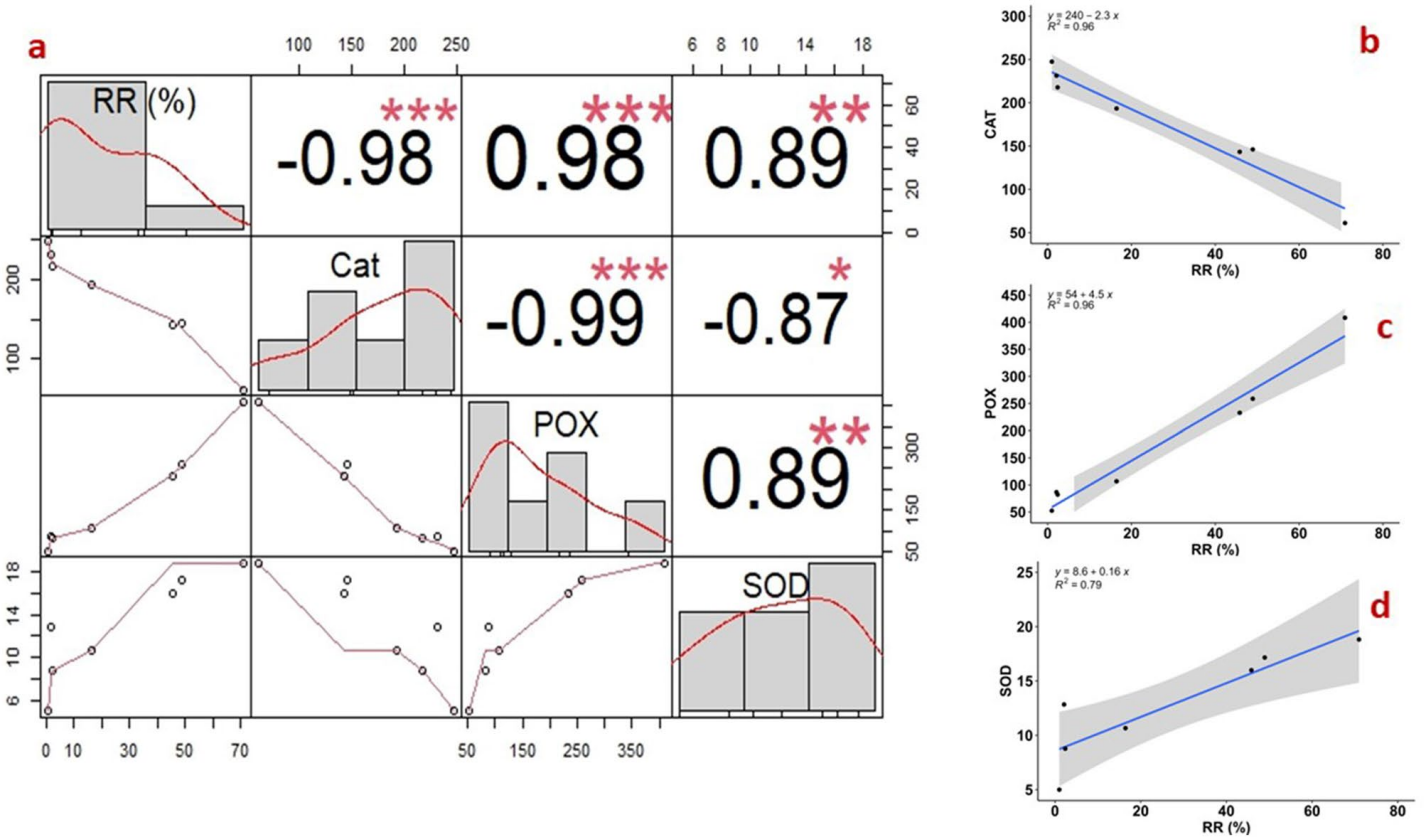
Correlation and regression analysis of resistance ratio (RR) with the antioxidant enzymes: SOD, POX, and CAT in *T. castaneum*. (**a**) Correlation of RR with SOD, POX, and CAT; (**b**–**d**) regression equation of RR with CAT, POX, and SOD, respectively. The shaded area in the regression analysis represents the 95% confidence interval. The R^2^ value is also mentioned. Signif. codes: 0 ‘***’ 0.001 ‘**’ 0.01 ‘*’ 0.05 ‘.’ 0.1 ‘’ 1.

The strength of the antioxidant enzymes and resistance level (RR) of different *T. castaneum* populations was analyzed through a Pearson’s correlation model (Fig. 3a). Our study highlighted the significant influence of phosphine exposure on CAT, POX, and SOD activities in all *T. castaneum* populations examined in this study. The values of Pearson’s correlation coefficients were positively linked with the resistant ratio factor of different *T. castaneum* populations for POX (r = 0.98, p < 0.001) and SOD (r = 0.89, p < 0.05) and observed significant variability. Conversely, the CAT activity was negatively associated with the RR factor with r = − 0.98 at p < 0.001 (Fig. 3a). The regression analysis used the resistant factor as an independent variable and the antioxidant enzymes as the dependent variable. The R^2^ values for POX, SOD, and CAT are recorded as 0.96, 0.79, and 0.96, respectively (Fig. 3b–d). Furthermore, the slopes are positively associated with SOD and POX, whereas CAT negatively negates with resistance ratio (Fig. 3). Our study found the susceptibility of populations increased with decreased SOD and POX activity.

**Figure 4:**
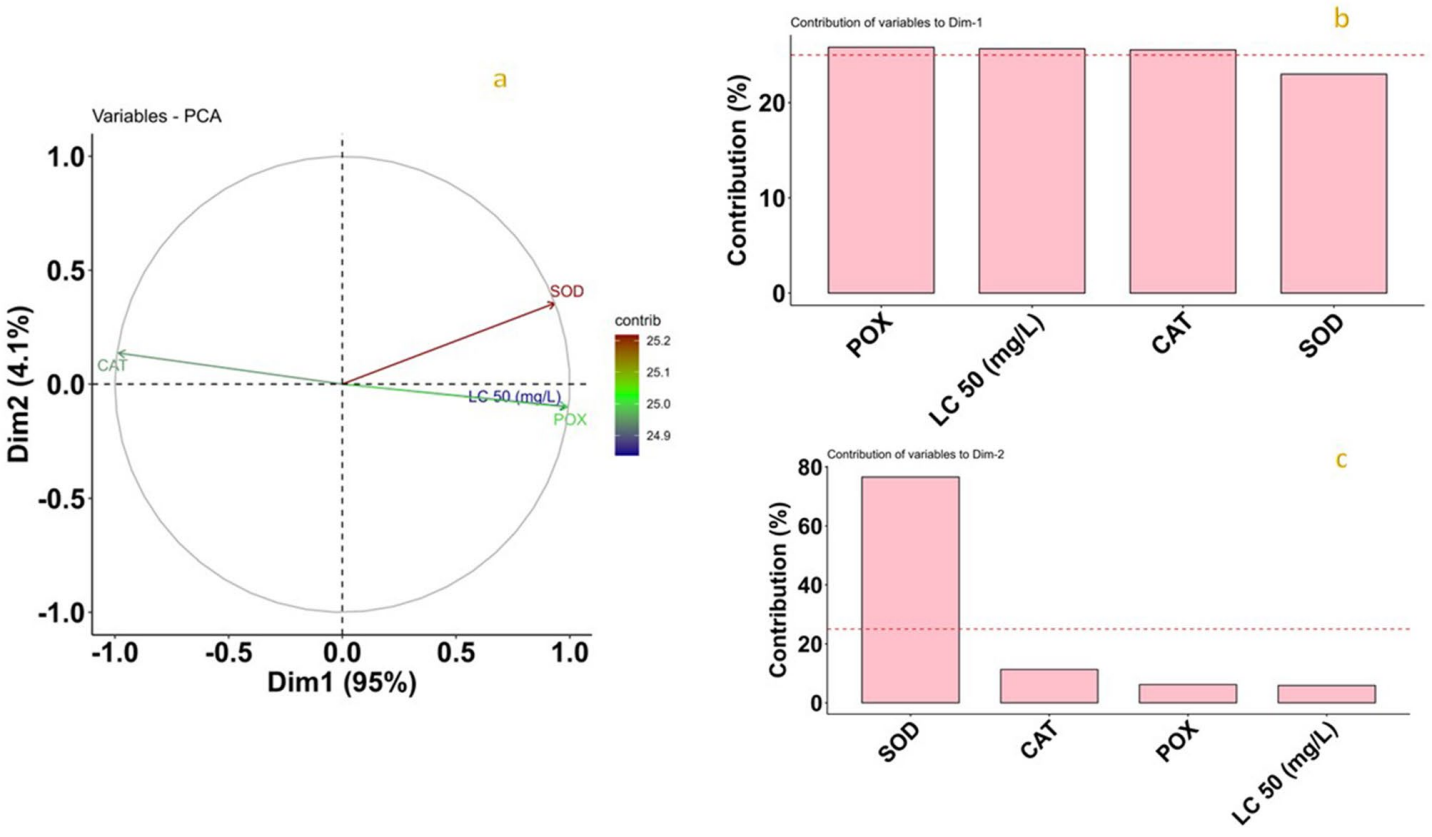
PCA biplot and contribution of studied traits to dimensions in *T. castaneum*. (**a**) PCA biplot, Presentation of four variables viz., LC50, CAT, POX, and SOD; (**b**,**c**) contribution of LC_50_, CAT, POX, and SOD to dimensions one and two, respectively; PC1 and PC2 are represented on the horizontal and vertical axes, with a cumulative variance of 95% and 4.1%, respectively.

PCA based on toxicity data and the specific activity of antioxidant enzymes indicated that the first principal component contributed 95% variation (Fig. 4a); the significant contributors are POX, CAT, and LC_50_. (Fig. 4b). The second dimension represents the 4.1% variation receiving contribution only from the SOD parameter (Fig. 4c). Our study revealed that the eigenvalue was 3.80 and 0.16 for dimensions one and two, respectively (Table 4). The traits POX, SOD, and LC_50_ were distributed with an acute angle, indicating a positive relationship, whereas the CAT showed a negative association with all the studied traits (Fig. 4). All the components having similar lengths symbolized a good amount of information to component contribution (Fig. 4).

**Table 4:** Eigenvalue, percentage of variance, and cumulative variance of PCA study.

Component	Eigenvalue	Percentage of variance	Cumula1tive percentage of variance
First	3.800	95.006	95.006
Second	0.164	4.121	99.128
Third	0.022	0.565	99.694
Fourth	0.0122	0.305	100.000

This table provides the percentage variance, and cumulative variance for each component of PCA output; the variability in eigen values presents the reduction in dimension.

**Figure 5:**
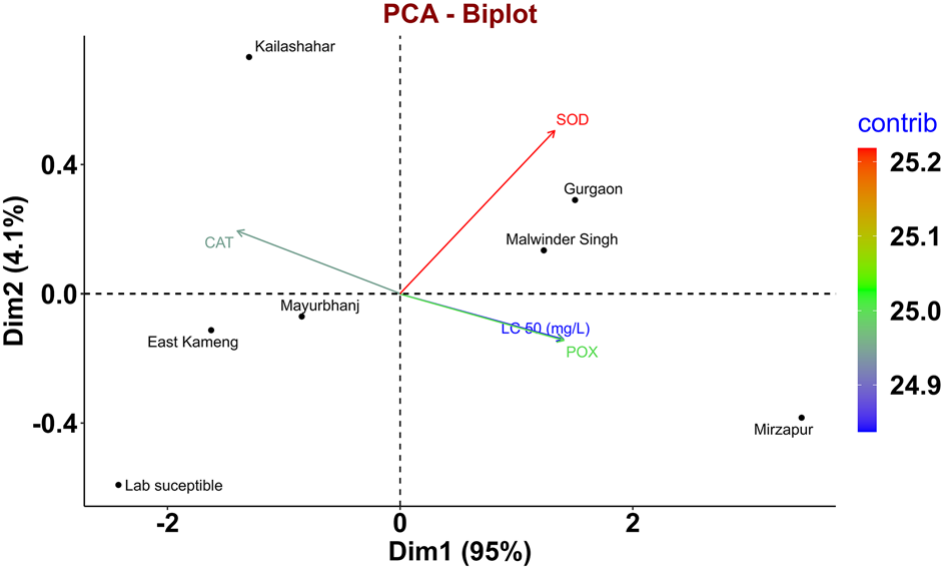
PCA analysis considering locations and all traits (LC_50,_ CAT, POX, and SOD). Visualization of correlation showing the influence of antioxidant enzymes on the LC_50_ of the populations used in this study; Black dots (●) in the figure represent active observations referring to different populations in the study. The distance of the variables from the center indicates the magnitude of influence; PC1 and PC2 are represented on the horizontal and vertical axes, with a cumulative variance of 95% and 4.1%, respectively.

The grouping of the population is depicted in Fig. 5. The least susceptible (Mirzapur) and most susceptible (Kailashahar) were opposite to each other are distinctly presented (Fig. 5). Malwinder Singh and Gurgaon populations showed lower susceptibility and were grouped in the biplot, corresponding with POX and SOD. Moreover, the biplot depicts the higher susceptible populations to phosphine, viz., East kameng and Mayurbhanj having lower LC_50_ was close to each other (Fig. 5). In addition, those populations were more closed to catalase depicting higher catalase activity (Fig. 3a).

## Discussion

Phosphine as a fumigant is extensively used to manage the stored grain pest worldwide, as it is cost-effective and leaves no harmful residue on commodities^26^. The present study gives the current phosphine susceptibility/resistance levels in contemporary field populations of *T. castaneum* collected across India. A differential susceptibility to phosphine as reported in this study (Table 2) had earlier been documented in key grain pests globally^8^. The occurrence of varying levels of susceptibility to phosphine has earlier been reported in *T. castaneum* from India^27^, in *Trogoderma granarium* from Pakistan^28,29^ and in S*itophilus zeamais* from Brazil^30^. Compared to an earlier report^31^, there has been a significant loss of susceptibility to phosphine implying the widespread prevalence of phosphine resistance in the contemporary field populations of *T. castaneum.*

The over-dependency upon phosphine and inappropriate fumigation practices might have triggered the build-up resistance to phosphine in key stored product insect pests across Asia, Africa, and South America^32–34^. Henceforth, this study revealed the burgeoning issue of phosphine administration in diverse storage conditions. The prevalence of phosphine resistance might be due to resistance genes in the population or by the selection pressure to phosphine stress^35^. The variability in the resistance ratio among the different populations within the same species, as observed in the study, might be due to their genetic diversity^13,36^ and distinct geographical territories^37^.

Our probit analysis found LC_50_ at 0.038 and 1.277 mg L^−1^ for the high and least susceptible populations, including Kailashahr and Mirzapur (Table 2). Compared to an earlier report, there have been consistent findings were observed regarding phosphine resistance (having lowest median dose was at 0.358 and 1.901 mg L^−1^ in the Gohana and Ajmeer) in the contemporary populations of *T. castaneum* in India^31^. However, an elevated range of LC_50_ was reported in *T. castaneum* populations from Pakistan^38^. In Oklahoma, USA, the study found that the LC_99_ value is 9.2 and 6.6 ppm for resistant *T. castaneum* and *R. dominica* populations, respectively^6^. However, after two decades, Opit et al.^7^ described that higher level of resistance in both the populations of Oklahoma, viz. *T. casataneum* (377.49 ppm) and *R. dominica* (3430.8 ppm). This highlights the escalation of resistance/diminishing susceptibility in the same population within a territory during a specific period, though our study outcomes are inconsistent with those findings.

The molecular analysis identified that the metabolic enzyme dihydrolipoamide dehydrogenase (DLD) was tightly linked to a phosphine resistance gene i.e., *rph2* in *T. castaneum* and other stored product insect pests^13^. However, recent investigations have highlighted the role of oxidative stress associated with antioxidant enzymes in phosphine toxicity^39^.

PH_3_, being a respiratory poison, induces oxidative damage by producing reactive oxygen species (oxygen-derived radicals), strongly contributing to phosphine toxicity^40^. Phosphine-induced oxidative stress was reported in many arthropods^41,42^, and mammals^43,44^. However, insects have a comprehensive antioxidant defence system to relieve oxidative stress caused by phosphine toxicity. Our results demonstrated increased activity of SOD and peroxidase with decreased susceptibility (Table 3). On contrary, the catalase activity was enhanced with increasing susceptibility (Table 3). SOD eliminates the ROS by the dismutation of superoxide anion to hydrogen peroxide through the catalytic process^45^. Our study found SOD activity was higher in Mirzapur (18.82 U mg^−1^ protein) followed by Gurgaon (17.16 U mg^−1^ protein) and Malwinder Singh (15.99 U mg^−1^ protein) population. Inversely, lower activity of SOD was found in higher susceptibility populations viz., East Kameng, Mayurbhanj, and kailashahar that are exhibiting lower LC_50_ values (Table 3)_._ Liu et al.^46^ demonstrated that the decreased activity of SOD in phosphine-susceptible insect populations supports our findings. However, phosphine susceptibility in stored insects is associated with enhanced oxidative metabolism under low oxygen (hypoxic) conditions^47^. On the contrary, the resistant population absorbs lesser phosphine to reduce oxygen metabolism^30^.

Catalase is an essential enzyme that detoxifies hydrogen peroxide to water. The catalase activity was positively linked with phosphine resistance in *T. castaneum*^48^. On the contrary, our findings revealed high catalase activity in susceptible populations such as the highly susceptible Kailashahar population (Table 3). We speculate that the reduced catalase activity in the least susceptible population of *T. castaneum* was probably attributed to decreased toxicant absorption. Bolter and Chefurka^49^ had earlier observed that the granary weevil, *Sitophilus granarius* population showing susceptibility to phosphine was having high catalase activity. The results of our study conform with this finding. In addition, a transcriptional study in *Drosophila melanogaster* revealed that the catalase gene was inhibited under phosphine stress conditions^46^. It curtails that phosphine is not directly inactivating its target enzymes but suppresses them by interfering with a complex signal transduction process^46^.

Though the peroxidase functions similarly to catalase, our findings revealed reduced activity of this enzyme in phosphine-susceptible populations (Table 3). Our results are consistent with that of Ranjith et al.^20^. On the contrary, Bolter and Chefurka^49^ observed reduced peroxidase activity in the phosphine-resistant *S. granarius* population. Similarly, the increased POX activity was noticed among *R. dominica* populations showing increased susceptibility to phosphine^41^. Yadav et al.^50^ observations on the activity of SOD and POX in *T. granarium* in response to phosphine also support our findings.

A significant positive correlation of SOD (r = 0.89) and POX (r = 0.98) with resistance ratio was observed in our study (Fig. 3a). Our results corroborated with an earlier report^42^. PCA biplot analysis (Fig. 4a) deduced the strong positive association of SOD and POX activities with the *T. castaneum* populations showing resistance to phosphine while catalase activity was negatively associated (Fig. 4b). The reduced activity of POX in susceptible populations could be due to the overproduction of H_2_O_2_ leading to cell oxidative damage. The increased activity of SOD and POX in resistant populations might have helped mitigate the negative impact of oxyradicals. Our result corroborated well with the earlier investigations by Yadav et al.^50^ and Ranjith et al.^20^ on *T. granarium* and *R. dominica,* respectively.

However, the cause of varied responses of antioxidant enzymes to phosphine toxicity in susceptible and resistant populations needs to be explored. Genomic and molecular investigations may elucidate in detail the cellular antioxidant mechanism in insects under xenobiotic stress.

## Conclusion

In conclusion, this study provided insight into the prevalence of phosphine resistance in the contemporary *T. castaneum* populations across India and associated antioxidant enzyme modulation. The study's indication of oxidative stress as a contributor to resistance presents an opportunity for heightened examination. An in-depth inquiry into the mechanistic intricacies of how these enzymes function in response to phosphine-induced stress, coupled with their putative involvement in detoxification pathways can substantially enrich the study's scientific rigor and conceptual coherence.

## Data Availability

All data have been provided in the manuscript.
